# Sequencing and characterization of the *Megachile strupigera* (Hymenoptera: Megachilidae) mitochondrial genome

**DOI:** 10.1080/23802359.2016.1166078

**Published:** 2016-04-12

**Authors:** Dunyuan Huang, Tianjuan Su, Bo He, Ping Gu, Ai-Ping Liang, Chaodong Zhu

**Affiliations:** a Key Laboratory of Zoological Systematics and Evolution (CAS), Institute of Zoology, Chinese Academy of Sciences, Beijing, China;; b Jiangxi Environmental Engineering Vocational College, Ganzhou, China

**Keywords:** Hymenoptera, mitochondrial genome, *Megachile strupigera*, Megachilidae, Megachilinae

## Abstract

The mitochondrial genome (mitogenome) of *Megachile strupigera* (Hymenoptera: Megachilidae: Megachilinae) was determined in our study. The sequenced region is 15,193 bp with an A + T content of 83.44%, including 13 protein-coding genes, two ribosomal RNAs and 19 transfer RNAs. All PCGs are initiated by typical ATN codons and stop with the complete termination codon TAA, except for *nad3* gene, which has an incomplete stop codon T. Bayesian method supported the monophyly of both Megachilidae and Apoidea. And within the Apoidea, Apidae and Megachilidae formed a sister clade to Colletidae.

Megachilidae is distributed nearly all over the world and comprises about 4000 species (Michener 2007; Ivanov et al. 2013). However, only one nearly complete mitogenome of Megachilidae has been reported (Zhang et al. 2015). Here, we present the mitogenome of *Megachile strupiger*a (Hymenoptera: Megachilidae), which was collected in Shadi Town, Gan County, Ganzhou City, Jiangxi Province, China (26°06′33.42″N, 114° 47′01.84″E). The type specimen was stored in the United States National Museum (accession number: 24884). And the specimen used in our experiment was primarily deposited in Institute of Zoology, Chinese Academy of Sciences and was identified carefully according to the description of the type specimen.

The nearly complete mitogenome of *M. strupiger*a is 15,193 bp in length with the A + T content of 83.4%. We were not able to amplify the region comprising part of *rrnS*, 3 tRNA genes (*trnA*, *trnM* and part of *Ile*), and the control region. This region has been proven difficult to sequence in other hymenopteran mitogenomes (Castro et al. 2006; Zhang et al. 2015). Analysis of the available sequence revealed the similar gene content, order and orientation found in other hymenopterans (Li et al. 2015; Wei et al. 2015). Additionally, 21 intergenic spacers (645 bp in total) and 6 overlapping regions (60 bp in total) are dispersed throughout the genome.

All 13 PCGs begin with typical ATN codons (five ATA, four ATT, one ATC and three ATG) and use standard termination codon TAA, except for *nad3*, which ends with a single T. In total, 19 of the 22 tRNA genes were sequenced. All the available tRNAs exhibit typical cloverleaf structures except for *trnS2*, whose dihydrouridine arm forms a simple loop, as reported in other Hymenopteran species (Wei et al. 2010; Huang et al. 2014). The *rrnL* and *rrnS* genes, with the A + T content of 82.2% and 80.3%, are located on the minority stand and separated by *trnV*, which is common in the mitogenomes of arthropods (Ye et al. 2010; Chai & Du 2012; Shi et al. 2015).

To construct a phylogenetic relationship of Hymenoptera, 41 complete hymenopteran mitogenomes were downloaded from GenBank. Besides, Hydaropsis longirostris was downloaded and used as the outgroup. The phylogenetic relationships based on nucleotide sequences of 13 PCGs using Bayesian inference method revealed the monophyly of both Megachilidae and Apoidea. And within the Apoidea, Megachilidae is the sister group of Apidae. What’s more, Apidae and Megachilidae formed a sister clade to Colletidae (Figure 1).

**Figure 1.**
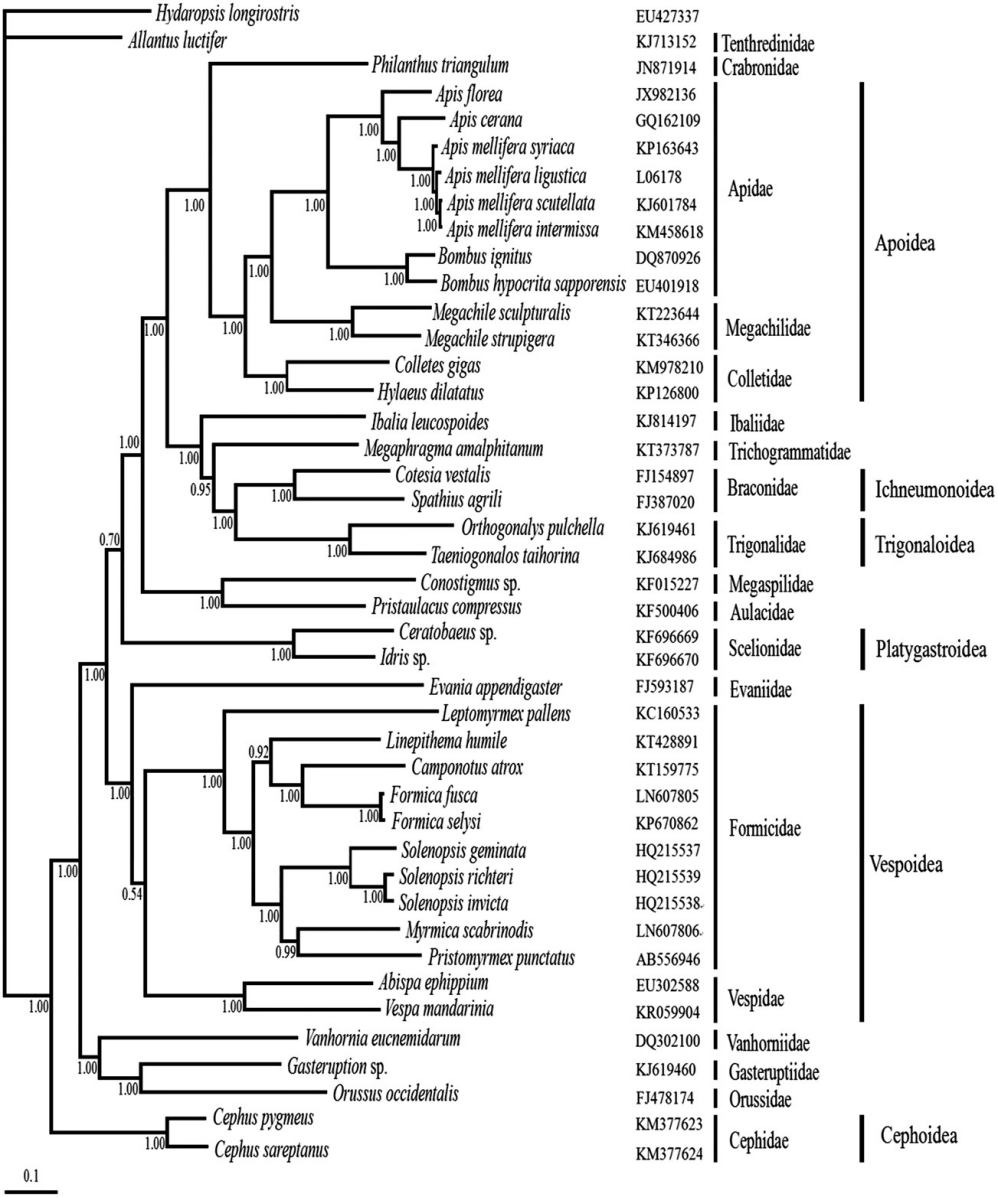
Inferred phylogenetic relationship among Hymenoptera based on nucleotide sequences of 13 mitochondrial PCGs using Bayesian inference. Each species used to generate the tree has a Genbank accession number on the right side of the corresponding scientific name. Number at each node show posterior probabilities

## Nucleotide sequence accession number

The mitogenome sequence of *M. strupigera* has been assigned GenBank accession number KT346366.
